# Fluoroquinolone resistance in bacteremic and low risk febrile neutropenic patients with cancer

**DOI:** 10.1186/s12885-015-1063-x

**Published:** 2015-02-06

**Authors:** Sheng Zhang, Qing Wang, Yun Ling, Xichun Hu

**Affiliations:** 1 Department of Medical Oncology, Fudan University Shanghai Cancer Center, Xuhui District, Shanghai, China; 2 Department of Oncology, Shanghai Medical College, Fudan University, 270 Dongan Road, 200032 Shanghai, China; 3 Department of Clinical Laboratory, The Affiliated Hospital of Qingdao University Medical College, Shinan, Qingdao, Shandong China; 4 Department of Clinical Laboratory, Shanghai Cancer Center, Fudan University, Shanghai, China

## Abstract

**Background:**

The low risk febrile neutropenic patients with Multinational Association for Supportive Care in Cancer (MASCC) score of more than 20 are recommended to be treated with fluoroquinolone-based oral treatment by the National Comprehensive Cancer Network (NCCN) guideline. This recommendation relies, at least partially, on the high sensitivity of the blood culture isolates to fluoroquinolone in clinical trials conducted in Western countries. Whether this also applies in middle or low income countries like China where antibiotic resistance is becoming prevalent recently has not been evaluated.

**Methods:**

All the positive blood culture results from January 2010 to December 2013 in the 2 large Chinese cancer centers were reviewed. The patients were included into the study with the following criteria: febrile neutropenia, solid tumor or lymphoma, MASCC score >20, positive blood cultures within two days of the onset of fever, and detailed treatment history.

**Results:**

A total of 38 patients were included in this analysis. Two patients had polymicrobial bacteremia (*Enterococcus faecalis* and *Flavimonas oryzihabitans*). Other isolates included coagulase-negative staphylococcus, micrococcal species, viridans streptococci, *Klebsiella pneumoniae*, and *Escherichia coli*. The majority of the monomicrobial isolates from these 36 patients was *Escherichia coli* (28 patients, 74%). Notably, in contrast to the high sensitivity to fluoroquinolone from blood culture of the low risk patients in previous reports in Westen countries, a very high drug resistance was observed: 13 out of 28 *Escherichia coli* isolates (46%) or 14 out of all 38 positive cultures (37%).

**Conclusion:**

The results warrant further validations in prospective clinical trials in countries where antibiotic resistance is prevalent to ensure appropriate antibiotic administration.

## Background

Febrile neutropenia, a serious complication commonly found in cancer patients receiving chemotherapy and/or radiotherapy, should be evaluated immediately. The patients with Multinational Association for Supportive Care in Cancer (MASCC) score more than 20 can be categorized as low risk [1]. The current American Society of Clinical Oncology (ASCO) guideline recommends immediate quinolone-based oral empiric antibiotic therapy for these patients [2,3].

This recommendation is based on several reasons: First, previous phase III trials as well as the observational studies have demonstrated the feasibility and success of oral quinolone-based empiric therapy for the low risk febrile neutropenic patients in some settings [4-9]. Indeed, the majority (>85%) patients can recover successfully with this treatment alone. Second, the culture results, especially the blood culture results showed consistently very low drug resistance to quinolone (~5%) which supported the routine use of quinolone-based antibiotic treatment [5,10].

Although the (ASCO) and National Comprehensive Cancer Network (NCCN) guidelines have been widely accepted and followed to guide daily practice for clinical oncologists worldwide, whether these guidelines could be indiscriminately generalized into the middle and low income countries like China where the antibiotics are easily accessible until recently is still debatable [11-13]. The antibiotic consumption per capita in China is nine times more than that in Western countries. The resultant antibiotic resistance has been increasingly recently, as in some other low and middle income countries [14-16].

We collected all positive blood culture results from January 2010 to December 2013 in two large cancer centers in China. In this study, the results from 38 patients whom can be categorized as low risk were analyzed.

## Methods

### FN definition

Neutropenia was defined as an absolute granulocyte count <0.5 × 10^9^ cells/L(<500/ul), or <1 × 10^9^ cells/L(<1,000/ul), expected to fall to <0.5 × 10^9^(<500/ul) within 24 hours, secondary to administration of chemotherapy and/or radiation within the last 30 days. Fever was defined as an oral (or tympanic)temperature >38.5 once, or ≥ two occasions (at least 1 hour apart) during the last 12-hour period, and suspected to be the result of infection.

All the positive blood cultures from January 2010 to December 2013 in the two large cancer centers (Shanghai Cancer Center, Fudan University; Cancer Center, The Affiliated Hospital of Qingdao University Medical College) were reviewed. The patients were first selected only if they met the febrile neutropenia criteria and were solid tumor or lymphoma patients. The patients were also selected if they have MASCC score >20. The detailed medical files of each patient were reviewed. Further selections include: The blood culture should be drawn within two days of the onset of fever to ensure the reliability of primary infection; Data regarding overall condition, use of antibiotics, development of serious complications were also reviewed. In order to ensure the reliability, only bacterial results from blood instead of those from urine or other sources were collected and analyzed.

The duration of antibiotics was measured as the number of days between the dates of the first and last doses of antibiotics. The duration of fever was measured from the date of presentation to date of resolution of fever (oral temperature <38 for a minimum of 24 hours). The duration of hospitalization was defined as the difference between the dates of admission and discharge from the hospital, inclusive of both days. The deaths within 30 days of the fever were viewed as early death and were reviewed to determine whether they were related to primary infection.

### Ethics statement

The independent ethics committees at the Shanghai Cancer Center of Fudan University and Cancer Center, The Affiliated Hospital of Qingdao University Medical College Sed this study. Written informed consent was waived because this is a retrospective study.

The study was undertaken in accordance with the ethical standards of the World Medical Association Declaration of Helsinki.

## Results

After careful review, a total of 38 patients with bacteremia whom can be categorized as low risk were included in this study. About a half of the patients were outpatients at fever onset. 71% patients had solid tumor, and the remaining patients had lymphoma. Detailed neutropenia and MASCC score profile were listed (Table 1). Grade 4 neutropenia was present in 37% of the patients; 76% had an MASCC score of 21 to 23, and 24% had a score of 24 to 26.

**Table 1:** **Demographic and clinical characteristics of patients (n = 38)**

Characteristic	No.	%
Male sex	21	55
Age, years		
Median	55	
Range	21-75	
Malignancy		
Lymphoma	11	29
Solid tumor	27	71
Status at fever onset		
Outpatient	16	42
Inpatient	22	58
Initial neutrophil count,/ul		
<499	14	37
500 to 999	21	73
MASCC score		
21	12	31
22	10	26
23	7	19
24	4	11
26	5	13

In microbiologically documented infections from blood culture, there were two patients with polymicrobial bacteremia (*Enterococcus faecalis* and *Flavimonas oryzihabitans*). These two patients responded to vancomycin-based antibiotic therapy and they subsequently recovered. The organisms isolated from other patients included coagulase-negative Staphylococcus, Micrococcus species, viridans group streptococci, *Klebsiella pneumoniae*, and *Escherichia coli* (Table 2). The majority of the monomicrobial isolates from these 36 patients were *Escherichia coli* (28 patients, 74%). There was no early death in the 8 patient with non-*Escherichia coli* bacteremia. The two early deaths which were bacteremia-related were both in the *Escherichia coli* group: two patients with bacteremia and development of septic shock died on day 11 and day 14, respectively. Remarkably, in contrast to the high sensitivity to fluoroquinolone from blood culture of the low risk patients in previous reports in westen countries, a very high drug resistance was observed: 13 out of 28 *Escherichia coli* bacteria (46%) or 14 out of all 38 positive culture (37%). The fluoroquinolone refers to ciprofloxacin or levofloxacin since these two agents were usually tested for the drug resistance.

**Table 2:** **Bloodstream infection details**

Organisms	No. of patients	Resistance*
Monomicrobial		
*Escherichia coli*	28	13
Micrococcus spp.	2	/
CoNS	3	/
Viridans streptococci	1	/
*Klebsiella pneumonia*	2	1
Ploymicrobial		
*Enterococcus faecalis* and		
*Flavimonas oryzihabitans*	2	/

*Abbreviation: CoNS* coagulase-negative Staphylococcus.

*Resistance to fluoroquinolone.

The median duration of antibiotic therapy was 15 days (range, 9-35 days). The median duration of hospitalization was 9.2 days (range, 5-28 days). The median fever duration was 4.3 days (range, 2–17 days). About 70% patients showed clinical improvement, while the remaining patients showed clinical deterioration. There were two early death which were related to the primary infection. About half of the patients received granulocyte colony stimulating factor (G-CSF). The quinolone based oral treatment was prescribed in 63% patients: 34% had ciprofloxacin and amoxicillin-clavulanate and 29% had quinolone alone. The remaining 37% patients took other oral antibiotics such as cefaclor (Table 3).

**Table 3:** **Treatment information and outcomes**

Outcomes	No.	%
Clinical deterioration	11	29
Survival at day 30	36	94
Further infection	11	29
Receiving G-CSF	18	47
Antibiotic duration, days		
Median	15	
Range	9-35	
Fever duration, days		
Median	4.3	
Range	2-17	
Duration of hospitalization, days		
Median	9.2	
Range	5-28	
Oral treatments		
Ciprofloxacin and amoxicillin-clavulanate	13	34
Quinolone alone	11	29
Other	14	37

## Discussion

Oral regimens for febrile neutropenia have largely relied on the activity of fluoroquinolone against Gram-negative bacteria, which may have been decreasing in recent years. Fluoroquinolone resistance in Gram-negative bacteria has been observed in febrile neutropenic patients in some countries [17-20]. However, this phenomenon was observed mainly in hematological patients and most patients cannot be categorized as low risk [19-21].

In the first and the only phase III, placebo-controlled, randomized trial for MASCC low risk febrile neutropenic patients, out of 19 Gram-negative bloodstream isolates causing primary infection among 333 patients, there was only one fluoroquinolone-resistant isolate (rate 5%) [5]. Similar low rate of fluoroquinolone resistance was also corroborated in the previous phase III trial in the patients who were not strictly categorized as low risk patients [10].

Our study here is one of the few studies which evaluated the low risk febrile neutropenic patients with blood culture results. The positive rate of blood culture for low risk febrile neutropenic patients is very low (about 5%), making collection and analyses of the data are very difficult [5]. Our result provided the largest data set regarding this topic. In contrast to the previously reported very low drug resistance to fluoroquinolone in the low risk febrile neutropenic patients mainly in Western countries, our study showed that a substantial resistance in this study, which may raise the caution when refer to recommendations in the NCCN guideline.

There are several possible reasons for the observed drug resistance to fluoroquinolone. First, there has always been one concern regarding the widely used prophylactic quinolone for the low risk febrile neutropenia. Antibacterial prophylaxis might select for microbial resistance, and conversely, resistance patterns may affect prophylactic efficacy [3,9,22,23]. Second, and more importantly, the situation with respect to overuse of antibiotics and antibiotic resistance in China is severe. The antibiotic consumption per capita in China is about nine times more than that in United States. In a study of resistance patterns of several common bacteria in China in 1999 and 2001, the mean prevalence of resistance among hospital-acquired infections was as high as 41% and that among community-acquired infections was 26% [24]. China also has the world’s most rapid growth rate of resistance (22% average growth in a study spanning 1994 to 2000) [14]. These factors taken together may contribute to the observed high fluoroquinolone resistance in this study.

The duration of hospitalization, antibiotic duration and time to defervescence are longer in our study compared with those reported in clinical trials. This is a common observation when comparing outcomes observed in carefully controlled clinical trials with those in general oncology practice [12]. Moreover, it can be also related to the nature of all patients who have bacteremia in this study and the drug resistance of isolated bacteria. The patients with bacteremia may need more time for the antibiotic treatment and hospitalization before they can recover.

Noteworthy, in our study, the prognosis of microbiologically documented infections was not severe. The mortality rate was acceptable, two early death out of 38 patients (rate, 5%) were viewed as primary infection-related. For the majority of the patients, the antibiotics were adjusted according to culture result or the empiric use by the physicians. The empirical use of the association of ceftriaxone and amikacin may have contributed to the overall fair outcome of infectious episodes. In addition, the frequent use of G-CSF (about half) in these patients may have improved outcomes because of reduced duration of neutropenia.

The results here raised a caution to generalize the recommendations from ASCO and NCCN guideline to the low and middle income countries like China [11]. In the recent years, ASCO has rapidly gained its recognition among oncologists in China. More and more educational programs have been introduced for the purpose of evidence-based medicine. However, most studies, especially the randomized phase III trials were conducted in developed countries [13]. The high prevalence of fluoroquinolone resistance among bacteria isolates from low risk febrile neutropenic patients in this study suggests different antibiotic coverage might be needed.

It was reported recently that the efficacy of once-daily, oral levofloxacin monotherapy was tested in a pilot study in Chinese patients with solid tumor and low-risk febrile neutropenia [25]. They demonstrated that oral levofloxacin could be effective for the patients. However, this study only has a limited sample size: the efficacy population was base on 41 patients per protocol. More importantly, blood culture results from these patients revealed there was no positive culture in this study. This suggests the patient population in this study might be different from that in our study. Only future large prospective, multicenter, phase III studies could establish the role of this oral antibiotic for low-risk, febrile neutropenic patients with solid tumor in areas where there is prevalent antibiotic resistance.

Our study has its intrinsic limitations of retrospective studies. The patient population from the two comprehensive cancer centers in China may not reflect patient polulations in community hospitals. It is also possible that patients who present to referral institutions may have more complex diseases. Due to the selection bias, the resistance to quinolone may have been over- or under-estimated, which need to be validated in the future prospective clinical trials.

## Conclusions

The high sensitivity of the blood culture isolates to fluoroquinolone from low risk febrile neutropenic patients is the basis for quinolone-based treatment which was recommended by NCCN guideline. A total of 38 low risk patients with positive blood cultures (this is the biggest data set regarding this topic.) were included in this analysis. A very high drug resistance was observed: 13 out of 28 *Escherichia coli* isolates (46%) or 14 out of all 38 positive cultures (37%). The results warrant further validations in prospective clinical trials in countries where antibiotic resistance is prevalent to ensure appropriate antibiotic administration.
